# Respiratory viruses associated with patients older than 50 years presenting with ILI in Senegal, 2009 to 2011

**DOI:** 10.1186/1471-2334-14-189

**Published:** 2014-04-08

**Authors:** Ndongo Dia, Vincent Richard, Davy Kiori, El Hadj Abdoul Khadir Cisse, Fatoumata Diène Sarr, Abdourahmane Faye, Déborah G Goudiaby, Ousmane M Diop, Mbayame N Niang

**Affiliations:** 1 Unit of Medical Virology, Institut Pasteur de Dakar, Unité de Virologie Médicale, Dakar, BP; 220, Dakar, Senegal; 2 Institut Pasteur de Dakar, Unité d’Epidémiologie des maladies infectieuses, Dakar, Sénégal

**Keywords:** Influenza, Respiratory virus, Elderly, Prevalence, Diversity

## Abstract

**Background:**

In Africa, especially in West Africa, studies about the prevalence and diversity of respiratory viruses (influenza and others) in elderly people are largely lacking. In studies done elsewhere, it is well established that older people, when compared with younger adults, are at greater risk of significant morbidity and mortality from complications arising from influenza. The main aim of this study was to determine the prevalence and the diversity of respiratory viruses associated with ILI cases in adults over 50 years old in Senegal.

**Methods:**

The recruitment period of this study was from January 2009 to December 2011. 232 patients aged 50 years and above presenting ILI cases were enrolled. Nasal-pharyngeal and/or oral pharyngeal swabs were collected from patients. RNA was extracted from 200 μl of each sample followed by a two-step real-time RT-PCR. The Anyplex™ II RV16 Detection kit was used for viral detection. The kit enabled the simultaneous detection of the presence of 16 respiratory viruses.

**Results:**

150 viruses were detected: influenza viruses (44.7%) and rhinoviruses (26.7%) were the most prevalent. We detected 13 human parainfluenza viruses (8.7%), 7 human respiratory syncytial viruses (4.7%), 6 coronaviruses (4%), 5 human metapneumoviruses (3.3%), 5 human adenoviruses (3.3%) and 1 human bocavirus (0.7%). 14 cases (6%) of dual virus infections and one triple viral detection case were encountered. 56 (56.6%) viruses detected were found in the 50-64 year old age group, 59 (76.6%; P < 0.001) from 65–74 year old age group and 35 (62.5%) were detected in the ≥75 year old age group. The viral co-infections were more frequent in the 65-74 age group (9/15).

**Conclusions:**

This pilot study demonstrates a variety of respiratory viruses in the elderly. It also highlights a high prevalence of these viruses in this age group. We speculate from these results that the impact of respiratory viruses other than influenza on the elderly has been considerably underestimated. A more exhaustive study seems necessary in order to provide a more complete picture of the burden of respiratory viruses on morbidity among adults over 50 years old in the sub-Saharan context.

## Background

Viral aetiology, prevalence and diversity data in people with influenza like illness (ILI) and/or acute respiratory illness (ARI) in Africa, (especially in West Africa), are scarce and often limited to the influenza viruses’ infection. Following the last influenza pandemic episode [1], few global and pediatric studies were conducted in some countries of the sub-region [2-4], and only a limited number of studies have described the etiology of ILI due to viruses including non-influenza respiratory virus [5-7]. However, no study has been conducted to describe the prevalence and the diversity of respiratory viruses (influenza and others) in West African elderly people. In studies done elsewhere, it is well established that older people, when compared with younger adults, are at greater risk of significant morbidity and mortality from complications arising from influenza [8,9]. For example in the United States alone, up to 40% of non-pneumonic lower respiratory illnesses in the elderly have been associated with respiratory viral infection [10], and an estimated 54,000 deaths annually have been attributed to the influenza and respiratory syncytial viruses (RSV) [11].

It should be highlighted that in Senegal the number of elderly people in consultation in healthcare centers for influenza like illness (ILI) is very low. Indeed, routine influenza monitoring in Senegal showed that samples from people above 50 years old represent only 3.7% of the total, over a 16 year surveillance period [4]. Some practices such as auto-medication and the use of traditional medicine to treat ILI largely explain this situation with the socio-economic situation being another contributing factor.

Thus the main aim of this study was to determine the prevalence and the diversity of respiratory viruses associated with ILI cases in adults over 50 years old.

## Methods

### Recruitment and samples

The recruitment period of this prospective observational study was from January 2009 to December 2011 inclusive. All 232 patients aged 50 years and above presenting with ILI during this period were enrolled in the study. It should be noted that samples were collected in the context of flu monitoring. An influenza sentinel surveillance system for outpatients with ILI was established in 1996 in Senegal and became part of the WHO Global Influenza Surveillance and Response System (GISRS). It is coordinated locally by the National Influenza Center (NIC) at the Institut Pasteur de Dakar.

Trained medical personnel were asked to screen all outpatients who were attended at the sentinel sites for signs and symptoms of ILI. The symptoms of influenza are similar to those arising from other viral respiratory pathogens. The inclusion criteria, according to the CDC case definition, were sudden onset of fever (≥38°C) with cough or sore throat fewer than 3 days in duration. Nasal-pharyngeal and/or oral-pharyngeal swabs were collected from each enrolled ILI case, placed in cryovials containing 3 ml of viral transport medium (Universal Transport Medium, COPAN Diagnostics Inc., Murrieta, CA, USA) and stored at 4°C on site. If nasal-pharyngeal and oral-pharyngeal swab specimens were collected from the same patient, both swabs were placed in the same cryovial. Upon arrival at the laboratory the specimens were separated into 3 aliquots for analyses. The first aliquot was used for molecular analysis for the detection of influenza viruses (real-time reverse transcription polymerase chain reaction or rRT-PCR detection), the second was used for influenza virus isolation, and the third was stored at -80°C for further analysis. The latter was used in the present study.

For each patient who met the case definition criteria, a form collecting demographic and clinical data was completed.The questions included information on date of enrollment and symptom onset, sex, age, clinical symptoms, previous treatments, vaccination status for influenza, and whether or not the patient was hospitalized.

### RNA extraction from clinical samples

Ribonucleic acid (RNA) extraction was performed from 200 μl of each sample using the QIAamp Viral RNA kit (QIAGEN, Valencia, CA, USA) according to the manufacturer’s instructions. Each RNA sample was eluted with 100 μl nuclease-free water before RNA quantification with a Nanodrop apparatus (NanoDrop Lite, Thermo Scientific).

### Detection of respiratory viruses

A two-step real-time RT-PCR was performed using the CFX96 Real-Time PCR Detection System (Bio-Rad).

### cDNA synthesis step

The RevertAid First Strand cDNA Synthesis Kit (Thermo Scientific) was used. First 1 ng of RNA was mixed with 1 μl of random hexamer primer and nuclease free water for a final volume of 10 μl. It was then incubated at 65°C for 5 minutes and immediately put on ice in order to remove the secondary structures in GC-rich RNA.

For the cDNA synthesis step, 4 μl of 5X reaction buffer, 1 μl of RNase inhibitor (20 u/μl), 2 μl of dNTP Mix (10 mM) and 1 μl of RevertAid M-MuLV Reverse Transcriptase (200 u/μl) were added and incubated for 5 minutes at 25°C followed by 60 minutes at 42°C and 70°C for 5 minutes. The cDNA product could be used directly for the next step (PCR amplification) or stored at -80°C until use.

### PCR detection

For viral detection, the Anyplex™ II RV16 Detection kit (Seegene) was used. The Kit enabled simultaneous detection of influenza A virus, influenza B virus, human respiratory syncytial virus A, human respiratory syncytial virus B, human adenovirus, human metapneumovirus, human coronavirus 229E, human coronavirus NL63, human coronavirus OC43, human parainfluenza virus -1, -2, -3, -4, human rhinovirus A/B/C, human enterovirus and human bocavirus.

Reactions are duplicated in two panels (A and B) for detection of the 16 viruses. The total reaction volume was 20 μl for each sample (for each panel), containing 4 μl 5X RV16 A (or 5X RV16 B), 4 μl of 8-MOP solution, 4 μl of 5X Anyplex PCR Master Mix (mix well by inverting 5 times) and 8 μl of cDNA product. PCR was assessed after 95°C for 15 minutes for transcriptase reverse enzyme inactivation, 50 cycles of 95°C for 30 seconds, 60°C for 60 seconds and 72°C for 30 seconds. 1 additional cycle of 55°C for 30 seconds was added for completion. The fluorescence is detected with a melting curve step, 55°C-85°C (5 seconds/0.5°C).

### Statistical analysis

Fisher’s exact test was used to verify whether the associated proportions were statistically supported and a p-value < 0.05 was considered statistically significant. We used the 50-64 year’ old group as the reference. The R.15.1 tool was used to perform the analyses.

### Ethical considerations

The Senegalese National Ethical Committee of the Ministry of Health approved the surveillance protocol as less than minimal risk research, and written consent forms were not required. Throughout the study, the database was shared with the Epidemiology Department at the Senegalese Ministry of Health and Prevention for appropriate public health action.

## Results

### Demographic and clinical information

A total of 232 patients above 50 years old were enrolled into the study, 129 (55.8%) were women and 102 (44.2%) were men (Table 1). Patients’ ages ranged from 51 to 97 years, with a mean age of 66 years. Ninety-nine (42.7%) enrolled patients were between 51 and 64 years old, 77 (33.2%) between 65 and 74 years old and 56 (24.1%) were 75 years old or older.

**Table 1:** Demographical, viral detection and clinical data

**Age groups**				
***Demographical data***	**50-64 y (n = 99)**	**65-74 y (n = 77)**	**≥75 (n = 56)**	**Total n (232)**
Mean age	56 y	70 y	81 y	66 y
Gender				
no. (%)				
Female	55 (55.5%)	41 (53.9%)	33 (58.9%)	129 (55.8%)
Male	44 (44.5%)	35 (46.1%)	23 (41.1%)	102 (44.2%)
***Viral detection rates***	***56 (56.6%)***	***59 (76.6%)***	***35 (62.5%)***	**150 (64.6%)**
***Clinical data***				
no. (%)				
Fever	89 (89.9%)	71 (93.4%)	49 (87.5%)	209 (90.5%)
Cough	70 (70.7%)	68 (89.4%)	48 (85.7%)	186 (80.5%)
Rhinitis	68 (68.7%)	67 (88.2%)	41(73.2%)	176 (76.2%)
Myalgia	35 (35.3%)	59 (77.6%)	32 (57.0%)	126 (54.5%)
Pharyngitis	26 (26.3%)	48 (63.2%)	28 (50.0%)	102 (44.2%)
Sore throat	41 (41.4%)	32 (42.1%)	23 (41.1%)	96 (41.4%)
Laryngitis	02 (2.0%)	20 (26.3%)	13 (23.2%)	35 (15.1%)
Headache	13 (13.1%)	08 (10.5%)	04 (7.1%)	25 (10.8%)
Dyspnea	02 (2.0%)	05 (6.6%)	04 (7.1%)	11 (4.8%)

Fever was the most reported clinical symptom, in 213 (92.2%; 213/232) of the enrolled patients, followed by cough (80.2%; 186/232), rhinitis (75.9%; 176/232), myalgia (53.9%; 125/232) and pharyngitis (44%; 102/232).

### Viral detection

In all, 132 (56.9%) out of the 232 patients were found to be infected with at least one of the viruses of interest. A total of 150 (64.6% of patients) viruses were detected. Of these viruses, Influenza viruses (44.7%; 67/150) and rhinoviruses (26.7%; 40/150) were the most prevalent viruses detected (Table 2). We detected 13 human parainfluenza viruses (3 PIV1, 4 PIV3, 6 PIV4) (8.7%), 7 human respiratory syncytial viruses (6 RSV A and 1 RSV B) (4.7%), 6 coronaviruses (4 coronaviruses NL63, one coronaviruses 229E and one coronavirus OC43) (4%), 5 human metapneumoviruses (3.3%), 5 human adenoviruses (3.3%) and one human bocavirus (0.7%).

**Table 2:** Viral etiology of influenza-like illness cases in adults aged above 50 years in Senegal during 2009-2011

	**Age groups**			
**Viral detection**	**50-64 y (n = 99)**	**65-74 y (n = 77)**	**≥75 (n = 56)**	**Total n (232)**
**Virus detected/n (%)**	**56 (56.6%)**	**59 (76.6%)**	**35 (62.5%)**	**150 (64.6%)**
Influenza A	24 (24.2%)	20 (26%)	8 (14.3%)	52 (22.4%)
Influenza B	4 (4.0%)	10 (13%)	1 (1.8%)	15 (6.5%)
RSV	2 (2.0%)	3 (3.9%)	2 (3.6%)	7 (3.0%)
Rhinovirus	12 (12.1%)	16 (20.8%)	12 (21.4%)	40 (17.2%)
Parainfluenza	3 (3.0%)	5 (6.5%)	5 (8.9%)	13 (5.6%)
Coronavirus	6 (6.0%)	0 (0.0%)	0 (0.0%)	6 (2.3%)
HMPV	3 (3.0%)	1 (1.3%)	1 (1.8%)	5 (2.2%)
Enterovirus	2 (2.0%)	3 (3.9%)	1 (1.8%)	6 (2.3%)
Adenovirus	0 (0.0%)	0 (0.0%)	5 (8.9%)	5 (2.2%)
Bocavirus	0 (0.0%)	1 (1.3%)	0 (0.0%)	1 (0.4%)
Codetections	2 (2.0%)	9 (11.7%)	4 (7.1%)	15 (6.5%)

A total of 14 cases (6%) of dual virus infections and one triple viral detection case were encountered. Influenza viruses (13 cases) and rhinoviruses (8 cases) were the most common type of virus found in samples with coinfections.

Regarding the number of viruses detected per age group, 56 (56.6%; 56/99) were from the 50-64 age group, 59 (76.6%; 59/77; P < 0.001) from the 65-74 year old age group and 35 (62.5%; 35/56) were detected in the older than 75 year old age group (Table 2). The viral co-infections are more frequent in the 65-74 year old age group (9/15) followed by the ≥75 years group (4/15).

Taking into account the clinical symptoms and viral detection, cough, rhinitis, pharyngitis or headache were in similar proportion in viral positive and non-positive patients: cough was observed in 79.4% of positive patients and 77% in the negative patients group (P = 0.17), rhinitis 79.4% and 67% (P = 0.04), pharyngitis in 45.8% and 42% (P = 0.74) respectively. In contrast myalgia symptoms are significantly higher among viral-positive patients: 76.3% versus 25% (P < 0.001).

**Figure 1:**
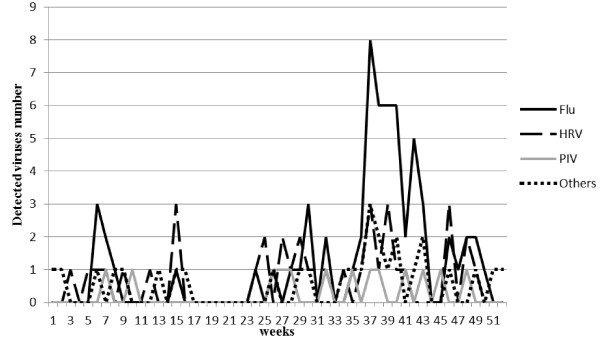
Weekly repartition of respiratory viral detection positives in patients older than 50 years in Senegal during 2009 and 2011 years.

The pattern of the virus detection throughout the study period is showed in the Figure 1. Influenza viruses (A and B) were mostly detected from July to August (between weeks 28 and 43), which corresponds to the rainy season in Senegal. A minor detection peak is also registered at the beginning of the year. Rhinoviruses and parainfluenza viruses showed homogeneous detection levels throughout the study period. Grouped, the remaining viruses seemed to have a similar temporal pattern to that of influenza viruses. The gap observed between weeks 18 and 22 correspond with a lack of samples from patients from our targeted age group.

## Discussion

The present study is the first description of the etiology of respiratory viruses associated with patients with ILI in a cohort of elderly people in the West African context. The results obtained showed that 132 samples of the study population out of 232 contained at least one of the targeted respiratory viruses.

The frequency of virus detection (56.9%) among the elderly with ILI in our study is consistent with that of several studies already conducted. Huo et al. (2012) [12], in a similar study in China detected at least one respiratory virus in 53% of patients 60 years old or older, and Munoz et al. (2000) [13] 49% in elders in a long term care facilities in Ontario during the 1998-1999 period. Hasman et al. (2009) [14] detected at least one respiratory virus in 68% patients in a study conducted in USA, without any precision about the ages of the 154 adults. In others studies frequencies are lower. For example Nicholson et al. (1997) [15] 43% (211/497) of viral detection among the elderly between 60 and 90 years of old, 36% (185/512) detected in the elderly over 65 years old in a study in China [16] and 22.2% in a recent study in Japan [17]. It is important to note that the technical approach used explains some discrepancies in rates of detection: primarily in their sensitivity and secondly in the number of targeted viruses. Alternatively differences in rates of detection could be due to true geographical differences in overall burden, differences in study populations (outpatients or hospitalized patients) and to the studies sample collection periods. Overall, the viral detection rate in the present study is very high as elderly people are often protected by pre-existing antibodies from previous illnesses, maybe illnesses suffered even decades back [18-20]. Indeed, because of pre-existing systemic and mucosal antibodies, elderly adults have been observed to have lower amounts of respiratory secretions and lower viral loads compared to children [10]. Consistent with this hypothesis, Falchi et al. (2011) [21] noted that the age distribution differed significantly between positive and negative patients, with positive patients being younger than negative patients (OR = 0.98, IC 0.95– 0.99; P = 0.0226).

Of the 150 viruses detected in the elderly, influenza A virus was the most common viral pathogen. Combined with influenza B viruses, influenza viruses represented 45% (67/150) of viruses detected. This influenza detection rate was expected as the enrollment of patients was directed towards patients with ILI. These results are in agreement with previous findings in the elderly [14,17,22]. Our study revealed a high detection rate of rhinoviruses (40/150; 26.7%). Rhinovirus is the most common respiratory pathogen in all age groups [23]. In a previous study, Nicholson et al. (1997) [15] showed that rhinoviruses were responsible for a greater disease burden (activities restriction, duration of illness) than that of influenza in elderly subjects representing 52% of detected viruses. In another study published by Greenberg (2002) [24], rhinovirus was the most prevalent pathogen (121 isolates; 53%) of the 231 identified in upper respiratory episodes. These findings are in concordance with the high rhinovirus detection rate in the present study.

With lower prevalence, PIV, RSV, HCoV, HMPV, enteroviruses, adenoviruses and bocaviruses were identified from elderly patient’ specimens and contributed collectively to 28.7% of all ILI cases in our study. These results show the high diversity of respiratory viruses circulating in the elderly population. This viral diversity supports previous results [10,12,24] and often in similar distributions with those of the present study.

Co-infections were relatively common in this study especially in the 65-74 years old age group (11.7%; 9/77). The rate found in this age group was in line with the findings of Hasman et al. (2009) [14] (11%) and Huo et al. (2012) [12], 11.7%. Huo and colleagues, in agreement with our results noted that co-infections were found most commonly in adults older than 60 years of age.

Focusing on clinical symptoms, with the exception of myalgia, our study showed no significant differences between viral-positive and viral- negative patients with ILI.

Viral circulation observed during the study period showed different patterns depending on the viral types. If we consider influenza viruses, we observed a circulation peak during the period starting in week 35 and ending in week 44. This period corresponds to the middle of the rainy season in Senegal. This result is further supported by a recent study conducted by Mbayame and colleagues [4]. These authors established clearly the seasonality of influenza viruses in Senegal after many years of surveillance with a regular circulation during the year and a peak in the middle of the rainy season (July-August-September). The slight peak of influenza observed at the beginning of the year (February) is the result of the shift caused by the recent pandemic episode. The pandemic occurred in early 2010 in Senegal with a peak in February [25]. Rhinoviruses showed a regular yearly circulation with peaks along the year corresponding to any rain season influence. The remaining respiratory viruses (PIV, RSV, HCoV, HMPV, enterovirus, adenovirus and bocavirus) were more likely associated with ILI peak during the rainy season. This co-circulation with influenza viruses was also seen in a previous pediatric study in Senegal [6]. Further studies (multiple year surveillance) are needed in order to properly define the temporal patterns of non-influenza virus circulation in Senegal.

Our study did have several limitations. The first weakness is the small number of samples treated in this study. A more exhaustive sampling would give a better representation of the different targeted viruses in the ILI cases among the elderly population in Senegal. Unfortunately after 16 years of influenza sentinel monitoring we noted that the number of elderly presenting at healthcare centers for ILI consultation is rather low compared to other age groups (children and young adults). The absence of nursing home services as in industrial countries, the use of traditional medicine (especially among the elderly) and economic constraints do not facilitate such studies in the West African context.

It is worth noting that this was a retrospective study, the database contained limited information on disease outcome and atypical clinical symptoms in ILI patients which were not reported. Thus the association between viral infections (or co-infections) and severe signs could not be established. As in previous studies it appears that co-infections were associated with more severe signs than mono-infections [26,27]. Without such data we could not measure the burden of targeted respiratory viruses in older patients with ILI. Another limitation is that our study is only focused on outpatient’ cases; it would be interesting to investigate hospitalized patient cases (severe cases). A final limitation was that the study included mainly one geographic location, Dakar, the capital city of Senegal.

## Conclusion

Despite the small number of samples included, the present pilot study demonstrates a variety of respiratory viruses in the elderly. It also highlights a high prevalence of these viruses in this cohort. From these results, it appears that the impact of respiratory viruses other than influenza was considerably underestimated. A more exhaustive study (increasing the number of elderly patients, with a better clinical picture and better documentation including disease outcomes, illness duration, hospitalizations etc.), relying on the new sentinel surveillance system (extension of sentinel sites in others geographical areas), seems necessary in order to provide a more complete picture of the burden of respiratory viruses on morbidity among adults over 50 years old in the sub-Saharan context.

## Abbreviations

ILI: Influenza-like illness; rRT-PCR: Real-time reverse transcription polymerase chain reaction; WHO: World Health Organization; cDNA: Complementary deoxyribonucleic acid; RNA: Ribonuleic acid; GISN: Global Influenza Surveillance Network; NIC: National Influenza Center; PIV: Parainfluenza virus; RSV: Respiratory syncytial virus; HCoV: Human coronavirus; HMPV: Human metapneumovirus.

## Competing interests

The authors declare that they have no competing interests.

## Authors’ contributions

The work presented here was carried out in collaboration between all authors. *MNN* and *OMD*, defined the research and revised the manuscript; *ND* performed and coordinated technical work, wrote the draft and revisions of the paper; *DK* performed the main technical part of this work; *EAKC* and *AF* participated in the technical work; *DG* and *EAKC* participated in data management and analysis; *VR* revised the manuscript and participated in the monitoring of the surveillance sites; *FDS* participated in the monitoring of the surveillance sites. All authors have contributed to, seen and approved the manuscript.

## Pre-publication history

The pre-publication history for this paper can be accessed here:

http://www.biomedcentral.com/1471-2334/14/189/prepub
